# Assessment of an Ultrasonic Water Stage Monitoring Sensor Operating in an Urban Stream

**DOI:** 10.3390/s21144689

**Published:** 2021-07-08

**Authors:** Yiannis Panagopoulos, Anastasios Papadopoulos, Georgios Poulis, Emmanouil Nikiforakis, Elias Dimitriou

**Affiliations:** 1 Hellenic Centre for Marine Research, Institute of Marine Biological Resources and Inland Waters, 19013 Anavissos Attikis, Greece; tpapa@hcmr.gr (A.P.); g.poulis@hcmr.gr (G.P.); elias@hcmr.gr (E.D.); 2 EXM P.C. (Ex Machina), Chalkidikis 89, 11855 Athens, Greece; manolis@exm.gr

**Keywords:** non-contact sensors, pressure transducer, rivers monitoring, statistical analysis, ultrasonic, water stage

## Abstract

The monitoring of the water stage in streams and rivers is essential for the sustainable management of water resources, particularly for the estimation of river discharges, the protection against floods and the design of hydraulic works. The Institute of Marine Biological Resources and Inland Waters of the Hellenic Centre for Marine Research (HCMR) has developed and operates automatic stations in rivers of Greece, which, apart from their monitoring role, offer opportunities for testing new monitoring equipment. This paper compares the performance of a new ultrasonic sensor, a non-contact water stage monitoring instrument, against a pressure transducer, both installed at the same location in an urban stream of the metropolitan area of Athens. The statistical and graph analysis of the almost one-year concurrent measurements from the two sensors revealed that stage differences never exceeded 7%, while the ultrasonic measurements were most of the time higher than the respective pressure transducer ones during the low flow conditions of the dry period and lower during the wet period of the year, when high flow events occurred. It is also remarkable that diurnal air temperature variations under stable hydrologic conditions had an impact on the measured stage from the ultrasonic sensor, which varied its stage measurements within a small but non-negligible range, while the pressure transducer did not practically fluctuate. Despite a slightly increased sensitivity of the ultrasonic sensor to meteorological conditions, the paper concludes that non-contact sensors for the monitoring of the water stage in rivers can be useful, especially where danger for possible damage of submersible instruments is increased.

## 1. Introduction

The water quantity and quality monitoring of surface water bodies is essential for the preservation of a healthy aquatic environment and the sustainable management of water resources [1], with transparency in data collection being crucial for the monitoring reliability [2,3]. Automatic telemetric instruments for surface water monitoring are gaining ground as they can provide early warning services, essential for pollution mitigation and preparation against extreme events [4,5]. Wireless technologies make it possible to connect to remote areas, enabling fast data transmission at high temporal resolution, allowing the detection of both short-term events and long-term changes, essential for environmental research [6,7]. The latest technological developments of in situ sensors and telecommunication protocols provide continuous data flows at low operational and maintenance costs [8,9].

Particularly for streams and rivers, water stage (or water level), the height or elevation of the water surface above an established datum plane, is also important for designing works (bridges, embankments and levees) or for protecting communities and human property from flood inundation. Stage is also used as the independent variable in a stage–discharge relation to compute discharges [10]. Stream stages are systematically monitored by automatic sensors, devices that automatically determine, or sense, the height of the water column from the water surface to the river bed. Various types of recording methods and associated instruments are available including the traditional method of a float being on the water surface inside a stilling well, and the newer methods utilizing pressure transducers and non-contact sensors [11].

Submersible pressure transducers are usually self-contained units having a transducer element and electronic circuitry and can be deployed directly in the stream. The sensor has to be installed into a fixed pipe to some level that is lower than the minimum water level or if possible directly above the river bed. The effect of the physical pressure, force per square unit of surface area, that is applied on the pressure sensor generates an output signal that is converted to a pressure measurement. In order to determine the hydrostatic pressure imposed by the height of the water column over the pressure sensor, the effect of atmospheric pressure has to be removed from the sensor readings. To accomplish this, a vented cable technology can be applied for measuring and compensating for atmospheric pressure [12]. In a vented or ‘relative’ sensor, through a small ventilation tube in the cable the atmospheric pressure is applied to the back of the sensor diaphragm and the pressure readings are adjusted to hydrostatic pressure measurements. In a non-vented or ‘absolute’ pressure sensor, such a vented cable is not connected to the device and the sensor measures all pressure forces detected by the strain gauge, including atmospheric pressure. In such a case, the local atmospheric pressure must be obtained from another device or nearby meteorological station in order to compensate for atmospheric pressure.

The main disadvantage of submersible pressure transducers is that they require relatively high levels of maintenance and could be damaged or destroyed by environmental conditions [13]. Being in contact with water, in the event of a flood, the debris in the river can sweep the sensor away. These sensors may be damaged or destroyed by lightning striking the water, by freezing temperatures, while they need calibration that usually requires the sensor to be sent back to the factory. Moreover, temperature variations may affect the accuracy of submersible units. To correct this effect, most of the new instruments have an integrated temperature sensor and by applying mathematical calculations in the sensor electronic circuitry they improve measurement accuracy [12].

On the other hand, recent developments using acoustic, radar and optical methods emerge with instrumentation that provides accuracy and convenience of measuring water surfaces without direct contact. The acoustic non-contact methodology for measuring the stage generally uses a high-frequency acoustic transducer that propagates a sound wave through the air perpendicular to the water surface. The reflected acoustic wave is received by the transducer, converted to an electrical signal and processed into a water–surface stage [12].

There are several advantages of non-contact sensors over sensors that require direct contact with water. Non-contact sensors allow for easy deployment and installation with the sensor attached to existing infrastructures, such as a bridge or structure directly over the water. Thus, the need to construct expensive stilling wells and fix a sensor under water is avoided. This is also the case with potential fouling, corrosion and damage from debris problems [14].

The disadvantage of measuring water stage with ultrasonic equipment is that the transmitted and reflected signals may be affected by obstacles such as snow, rain or dust, by turbulent water surfaces, and even by air turbulence along the sound path. Moreover, sound speed depends on the temperature and to a lesser degree on the relative humidity and the density of the atmospheric air circulating between the sensor position and the water surface, which in turn affects the accuracy in distance measurement [15,16]. However, with compensating for air temperature and relative humidity, limitations are greatly reduced. Many manufacturers have temperature compensation built into their devices, with the use of internal temperature sensors, enabling the improvement of the accuracy of the ultrasonic sensors [17].

Within the framework of the European water legislation [18], the Institute of Marine Biological Resources and Inland Waters (IMBRIW) of the Hellenic Centre for Marine Research (HCMR) is in charge of coordinating the national surface water monitoring program in Greece [19,20], comprised of systematic water sampling and laboratory analyses for water quality. To further contribute to the national monitoring of surface water bodies, it has identified areas where monitoring needs are more intense and would be benefited by the installation and operation of automatic telemetric stations [21]. Since 2019, the Institute has established and operates numerous automatic stations measuring in the real time water stage and physicochemical water parameters across 11 rivers in the continental Greece [22]. Among the installed stations there is an urban river site where water stage is not only measured by a submersible pressure transducer embedded in the multiparameter sonde of the automatic station, but at the same time by a non-contact ultrasonic sensor. The double monitoring of the water stage was chosen for research purposes within the framework of the research project OpenELIoT [23] but was selected at this site in particular due to the urban environment and the potential consequences in the case of river overflowing. It offers a great opportunity for useful comparisons between the two sensor types allowing us to draw conclusions regarding a possible superiority of one type over the other and decide whether a non-contact water stage sensor of tolerable cost can be of wider adoption for reliable monitoring of rivers in the future.

## 2. Materials and Methods

**Figure 1:**
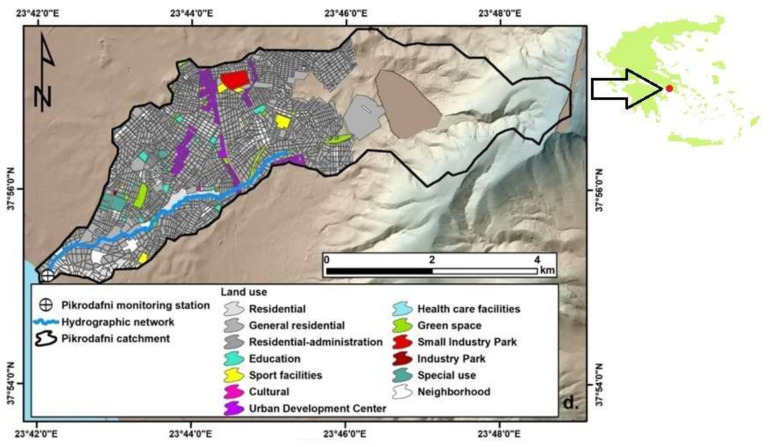
(From Mentzafou et al. [24].) The Pikrodafni urbanized catchment and the homonymous stream within the metropolitan area of Athens, Attica region, Greece.

The river site of interest is located near the mouth (37.922411, 23.700816) of Pikrodafni stream, a surface water body with a highly urbanized catchment [24] and a partially channelized course within the metropolitan area of Athens, Greece (Figure 1). As a result of the relevant pressures, increased values of nitrates, polycyclic aromatic hydrocarbons and total coliforms have been reported [25] and the biological communities (riparian vegetation, fish, birds and macroinvertebrates) exhibit low diversity and poor structure [26].

The automatic station’s main component is the sonde In-Situ Aqua TROLL 500 [27] measuring in real-time four physicochemical parameters and the water stage, which is important for evaluating the water quantity conditions in the river, especially with a focus on low flow (drought) and high flow (flood) conditions. Water stage is in practice measured with a piezometric water sensor, a submersible pressure transducer embedded within the sonde with a vented cable for atmospheric pressure compensation. Once the hydrostatic pressure values have been obtained, the water depth *h* can be calculated by using the hydrostatic equation *p* = *ρ* ∙ *g* ∙ *h*, where *p* is the hydrostatic pressure, *ρ* is the water density and *g* is the gravitational acceleration. In the internal calculations of the sonde, the water density was set to 1000 kg/m^3^ and the gravitational acceleration was set to the conventional standard value of 9.81 m/s^2^.

Data recording with the AT500 started in late 2019, while for the needs of the data quality checks applied, data were subject to a two-stage quality control procedure before their online dissemination. As a first step, algorithms apply plausibility tests to observations with a pass/fail criterion based on predetermined allowable data ranges and variability to detect possibly erroneous values [28,29]. As described in detail in Panagopoulos et al. [22], a range test was first performed in order to identify values outside the expected natural limits of each variable, considering the instruments limits and the physical ranges of the corresponding variable. For example, the temperature allowable range was set between 0 and 30 °C, pH between 5 and 10 and dissolved oxygen between 4 and 11 mg/L [22]. A water stage that is more site-dependent is usually considered to range normally within 0–5 m above the river bed. A test of extreme values (extreme value test) follows by marking the greatest and smallest values within the entire data set, and a test of extreme differences of successive values within the data set (extreme difference test) also follows, which indicates the largest and smallest absolute changes of consecutive observations. Finally, the data quality check included a persistence test, which examined whether or not a variable had stopped varying with time, indicating a non-response of the sensor to changes in the values of the variable (stuck value test). In a second stage suspicious values were labeled using specific flags and examined through graphs to judge whether these measurements represent extreme natural phenomena and should be integrated in the entire data series without labeling or whether they are of poor quality and have to remain flagged. So far, the two-stage quality control procedure for the parameter of the water stage has shown that the recording does not meet any particular problems at any installed Aqua Troll instrument, including the Pikrodafni station of the present analysis [22].

At the same location of the Pikrodafni stream, for the measurement of the water stage, an HRXL-MaxSonar-WR ultrasonic sensor was installed, which is an in-air, non-contact object detection and ranging sensor, not affected by the color or other visual characteristics of the detected object (water in particular) [30]. The ultrasonic sensor was mounted on a bracket perpendicular to the water and emits pulses of a high frequency acoustic wave perpendicular to the water surface. The transmitted pulse travels towards the water surface was reflected and travelled back to the sensor. The sensor measured the round-trip time of the pulse and the distance from the sensor to water surface can be calculated by using the relationship *d* = (*c* ∙ *t*)/2, where *d* is the distance, *t* is the elapsed time and *c* is the speed of sound. Each time a HRXL-MaxSonar-WR sensor takes a distance reading, it calibrates itself. If the temperature, humidity or applied voltage changes during operation, the sensor will continue to function normally over the rated temperature range while applying compensation for changes caused by temperature and voltage. Since sound speed depends on air temperature and to a lesser degree on relative humidity, the HRXL-MaxSonar-WR sensor was equipped with an internal temperature sensor, which allows the sensor to apply compensation for the speed of sound changes. Moreover, the HRXL-MaxSonar-WR sensor performed an operating-voltage compensation for improved accuracy enabling superior rejection of outside noise sources and featured 1-mm resolution. To calculate the water stage, the ultrasonic measured values (distances from the sensor to water) were subtracted from the stable distance of the sensor from the river bed measured at installation.

**Figure 2:**
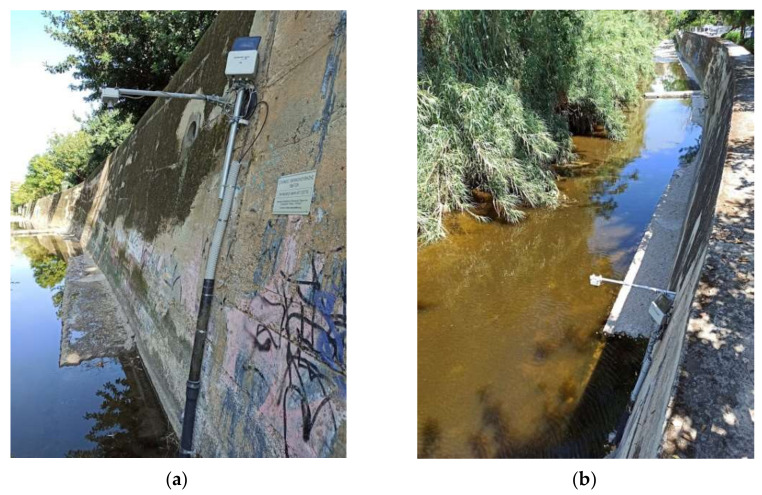
Two views (**a,b**) of the installation of the Hellenic Centre for Marine Research (HCMR) multiparameter sonde AT500 (including the pressure transducer) within the pipe attached to the wall and the ultrasonic sensor at the edge of the mounting bracket attached to the wall for water stage monitoring at the Pikordafni stream in the Attica region.

Similarly, with the sonde, the mounting bracket of the ultrasonic sensor was attached to the masonry of the demarcated Pikrodafni stream. Figure 2 shows the installation site of the ultrasonic sensor and the pipe within which the multiparameter sonde AT500 was installed.

The data from the Pikrodafni sensors are transferred telemetrically (via GPRS) to a server at time intervals ranging from 10 min to 1 h (10 min was preferred here in order to have the largest possible dataset), they are stored in databases and are published on the OpenELIoT [23] project platform and on an HCMR central visualization platform [31], which combines all stations of the new automatic monitoring network [22]. It is worth mentioning that the data logger’s clock is set to standard time (UTC) to avoid confusion during the transition to or from daylight saving time.

For the needs of the present study, we used the whole water stage data series from the two sensors that are available for a common period of time. Thus, the water stage data used in the analysis were from 17 June 2020 until 27–February 2021 at a 10-min interval. This resulted to a large dataset containing more than 23,000 records of the water stage from the pressure transducer and the ultrasonic sensor respectively. Although the data did not cover a whole calendar year, they covered the largest parts of both dry (May to September) and wet (October to April) periods. Thus, they included a quite long period of low flows and a representative period within winter with stage/flow peaks, respectively.

It is worth mentioning that the instrument AT500 functions using in the internal calculations for the water density the constant value of 1000 kg/m^3^ and for the gravitational acceleration the conventional standard value of 9.81 m/s^2^. Nevertheless, the water density depends on water temperature and the gravitational acceleration depends on latitude. Specifically, water density varies as a function of water temperature $t_{w}$ and this dependence can be expressed in terms of the international temperature scale of 1990 (ITS-90) [32] as:

(1) $$\rho =999.97495\cdot \left[1-\frac{{\left(t_{w}-3.983035\right)}^{2}\cdot \left(t_{w}+301.797\right)}{522528.9\cdot \left(t_{w}+69.34881\right)}\right]$$

In addition, following the World Geodetic System 1984 (WSG 84) the numerical form (DMA [33,34]) of gravitational acceleration is:

(2) $$g=9.7803267714\cdot \frac{1+0.001931851381639\cdot \sin^{2}\varphi }{\sqrt{1-0.0066943999013\cdot \sin^{2}\varphi \;}\;}$$

where *φ* is the latitude.

To test the accuracy of the internal calculations, we recalculated the water stage values using the most accurate expressions of the terms of the hydrostatic equation. We found a close match between the modified and the AT500 original water stage values exhibiting a high correlation (r^2^ = 0.99). The differences between the modified and the AT500 original water stage values range between 0.1 and 0.5%, which fall within the accuracy limits of the device. Therefore, in the analysis we decided to proceed with the AT500 original measurements.

Air temperature and water temperature data at 10-min intervals were also used in the analysis from the nearby meteorological station and the water temperature sensor of the multiparameter sonde, respectively. For analyzing the time-series data we used the Minitab statistical software [35] and the Microsoft Office Excel. We applied regressions between the subhourly data series from the two sensor types, calculated the percentage deviations of the pressure transducer stages from the ultrasonic sensor ones, applied correlations of the deviations and the stage data with air and water temperature data and we calculated statistics of the stage time-series and temperatures. To further investigate the main interrelationships, we also visualized fluctuations of water stages, their differences between the two sensor types, and temperatures, from selected shorter time-periods representative of the dry and wet periods.

## 3. Results

**Figure 3:**
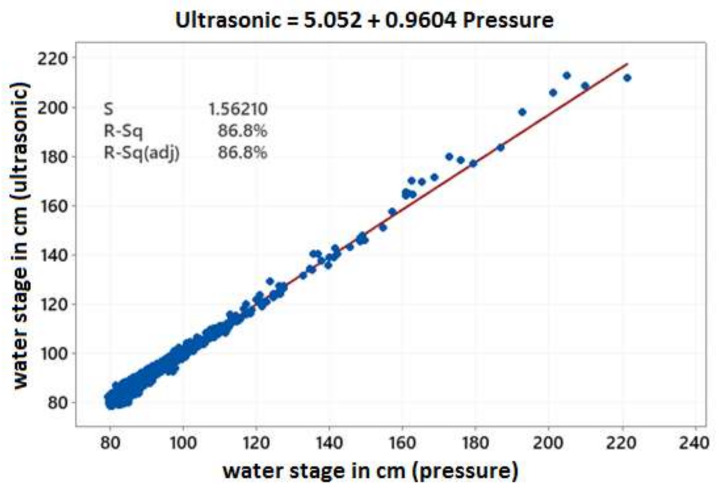
Correlation between water stages (in cm) measured with the pressure transducer and the ultrasonic sensor at Pikrodafni stream.

A direct correlation of the entire pressure transducer (AT500) and ultrasonic water stage data series was first applied to obtain a general overview of their relationship. As shown in Figure 3, the correlation was strong revealing that the sensors did not deviate considerably from each other at any level of the water stage recorded within the 9-month period with available data. Water stage minima were consistently in agreement, at around 80–100 cm, and could rise up to 200–220 cm during high flow episodes.

A statistical analysis with the Minitab software package calculated the means, standard deviations, minimum, maximum and the 1st, 2nd (median) and 3rd quartiles of the stage data and the deviations. The results are summarized in Table 1. As far as water stages are concerned, all parameters were comparable showing a similar spread of the recorded from the two sensors values. The deviations were calculated as the percentage differences of the pressure transducer values (p) from the ultrasonic sensor values (u) keeping as reference the first: 100 × (u − p)/p. It is worth noting that since less than 25% of the deviation data values were negative (Q1 for deviations is positive in Table 1), the majority of the ultrasonic measurements were greater than the respective pressure transducer ones.

**Figure 4:**
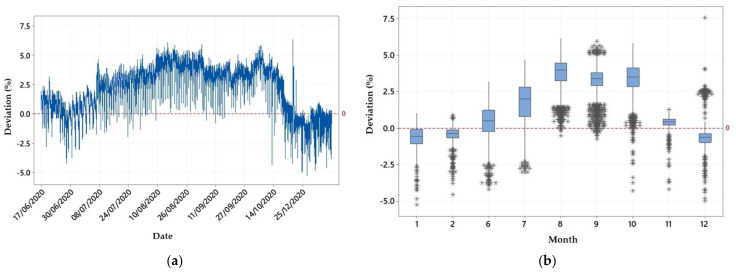
Percentage deviations of the measured pressure transducer water stages from the ultrasonic stages at Pikrodafni stream: (**a**) throughout the common period with available data from both sensors and (**b**) per month (nine months of available data).

The water stage deviations between the two sensors were also plotted with the date in the diagram of Figure 4a, while in Figure 4b they were plotted for each month with available data (nine months in total). As shown in Figure 4a, absolute deviations were never greater than 7% and actually from Table 1 the maximum deviation was 6.1349%. However, there was a clear tendency of the ultrasonic sensor to record a higher water stage than the pressure transducer sensor in the greatest part of the period with available data. In particular, there were positive deviations (higher ultrasonic recordings) throughout the time-period, but especially during the driest period of the year, from July to September, the vast majority of deviations were above zero (Figure 4a,b). This is also the case, but to a lesser extent, with June and October. Thus, within most of the dry period of the year, when rare or no high flow events occurred at Pikrodafni stream, the water stage from the multiparameter sonde was lower than the auxiliary recording from the ultrasonic sensor. On the other hand, during all months of the wet period with available data (November 2020–February 2021) deviations interchanged between the above and below the zero level with majority of them being negative.

With the Minitab software package, the Pearson correlation coefficients (Pearson’s *r)* between all the parameters of interest were then calculated, namely, water stages from the two sensors, deviations (%) of the ultrasonic stages from the pressure transducer ones, air temperature and water temperature. The correlation results are summarized in Table 2.

Obviously, the maximum correlation coefficient was observed for the two stage data series (pressure and ultrasonic) that revealed a covariation of the water stage, as has already been implied by Figure 3. This is the case for the two time-series of temperature showing that water temperature generally followed the fluctuation of air temperature (coefficient 0.958 in Table 2). For all other parameters, correlation coefficients were rather weak, with some of them being close enough to zero. For example, the % deviation was not correlated with water stages, thus, deviation magnitude could not be associated with the magnitude of water levels in the river. Similarly, both air and water temperatures did not seem to have any remarkable correlation with the absolute stages recorded (coefficients in Table 2 between −0.330 and −0.062), with the negative signs here being simply resulted from the fact that high temperatures in the dry period were obviously associated with low flows and river water stages. On the other hand, it seems that a much stronger correlation existed between water and air temperatures and the percentage deviations of stages from the two sensor types, being around 0.7 (see Table 2). The positive signs reveal a positive correlation, which may imply that highly positive deviations (ultrasonic stage measurements being higher than pressure measurements) were mostly associated with high temperatures, possibly in the dry period of the year when normal flow conditions predominated, while during periods of temperature decline the two sensors data became closer, resulting in lower percentage deviations.

**Figure 5:**
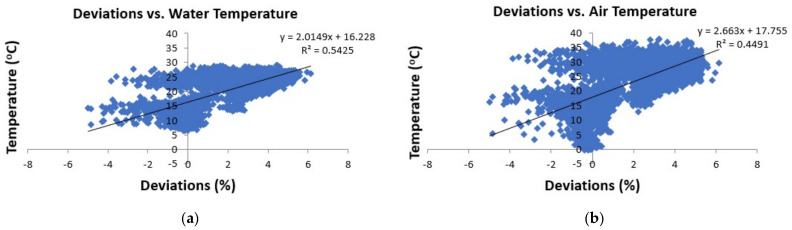
Percentage deviations of the pressure transducer water stages from the ultrasonic stages at Pikrodafni stream vs. (**a**) water temperature and (**b**) air temperature.

Indeed, by creating the correlation graphs of Figure 5 between % deviations and temperatures, the positive nature of the correlation was confirmed. We see that either with a water or air temperature increase the sign of deviation shifted from negative to positive. There were two big clouds of data-pairs, one in the negative and the other in the positive side of deviations (*x* axis); however, the majority of the 10-min stage deviations were lying in the positive side. Moreover, the positive deviations (pressure stages being lower than the ultrasonic stages) reached a higher maximum between 6 and 7% (maximum 6.13% in Table 1) than the negative ones which had their lowest at around −5% (5% in absolute terms, minimum −5.26% in Table 1).

**Figure 6:**
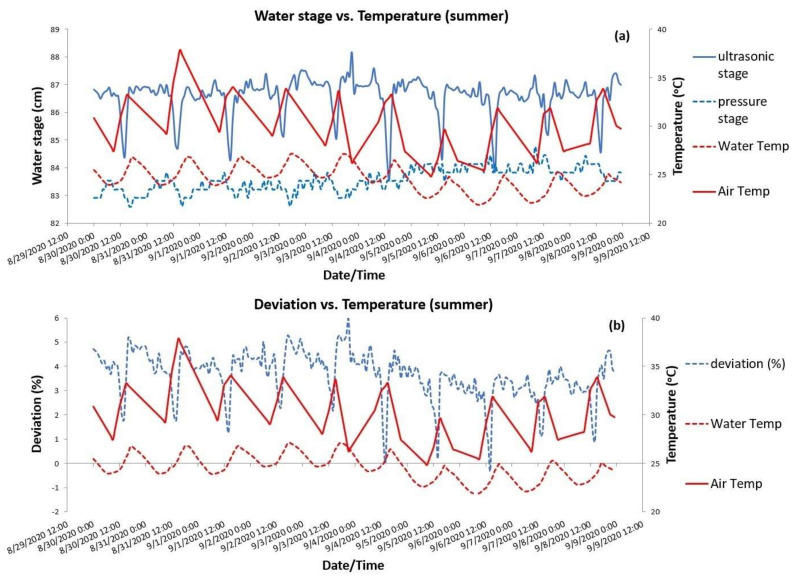
A selected 10-d summer period of observation at Pikrodafni stream: (**a**) ultrasonic and pressure water stages vs. temperatures and (**b**) deviations (%) of pressure stages from ultrasonic stages vs. temperatures.

To shed light on the phenomenon, two shorter periods of a few days were inspected with regard to the fluctuation of all the parameters of interest. Figure 6a depicts the ultrasonic and pressure stages fluctuation within a 10-d period in August-September, along with the fluctuation of water and air temperatures occurring at the same period. First of all, it can be noted that, as expected, water temperature was consistently lower than air temperature. Actually, by inspecting closely the graph, water temperature variation follows air temperature fluctuations with a 2-h response time. Ultrasonic water stage fluctuated within a 4-cm range, from 84 up to 88 cm, during the 10-d period of analysis when no rainfall occurred. On the other hand, the pressure transducer stages fluctuation was narrower between 83 and 85 cm, always lower than the ultrasonic stages. What is of particular interest is how the ultrasonic stages fluctuated within the rather small 4-cm stage range when no rainfall and significant runoff had occurred for days. It is shown that the ultrasonic water stage reductions coincided with the air temperature peaks. Thus, at noon or afternoon when temperatures reached their maximums above 30 °C, the ultrasonic sensor was influenced and the recorded stage declined by 2–3 cm.

As the pressure stages did not strictly follow the same pattern and given that their fluctuations were lying within a smaller range, the produced deviations between the two sensors were attributed to the ultrasonic sensor instability. In fact, temperature peaks caused the least difference in stages resulting in close to zero deviations but during the night and morning times when temperature was lower, deviations approached their positive maximums, close to 6% as shown in the bottom graph of Figure 6b.

**Figure 7:**
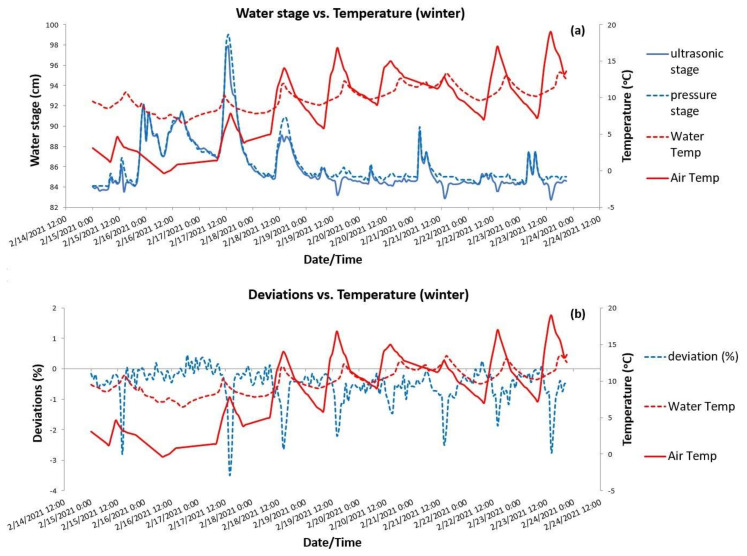
A selected 10-d winter period of observation at Pikrodafni stream: (**a**) ultrasonic and pressure water stages vs. temperatures and (**b**) deviations (%) of pressure stages from ultrasonic stages vs. temperatures.

A similar double graph was created in Figure 7 for a 10-d period in February. As being in the winter season, stage peaks occurred and were alternated with low flow conditions. First of all, air temperatures ranged between 0 and 20 °C, with water temperature varying much less and fluctuating within the smaller range of 8–13 °C. From February 20 the water temperature changes followed atmospheric ones with a response time of almost 4–5 h. The graph shows an almost perfect agreement between the two stage recorders, which measured water stage with negligible differences (Figure 7a). Thus, the deviations calculated did not exceed the level of −3% as shown in the bottom graph (Figure 7b). However, although they were small, all deviation peaks occurred when air temperature was peaking, with a negative sign due to the (slightly) lower ultrasonic measurements compared to the pressure ones. Excluding the dates with stage peaks, which were obviously caused by increased streamflow, the no-peak hydrologic conditions occurred in 19–20 and 22–23 February revealed a similar behavior of the ultrasonic sensor over air temperature with the behavior observed in the summer period of Figure 6a. Simultaneously with air temperature highs, ultrasonic stages were on their lows, creating observable gaps with the corresponding pressure stages. Thus, even the winter graph of Figure 7a confirms an existing sensitivity of the ultrasonic sensor to air temperature under stable hydrologic conditions. This sensitivity was however, smaller in winter than in summer, in absolute terms. Moreover, while ultrasonic stages were mostly superior in the dry period, they appeared to be slightly inferior in the wet period compared to the stages from the pressure transducer.

## 4. Discussion and Conclusions

The data and statistics presented in this paper were based on the measured water stage in the urban Pikrodafni stream of the metropolitan area of Athens from an ultrasonic sensor and a submersible pressure transducer. The data can be directly comparable since the sensors were located at the same point, which was close to the stream outlet to the sea, while they both counteracted or corrected the undesired influence of the meteorological conditions. Specifically, the ultrasonic sensor had an internal temperature sensor for air temperature compensation and the pressure transducer had a vented cable for barometric pressure compensation.

From the 9-month concurrent measurements, it was clearly shown that water stages from the two sensors had strong covariance both during the wet and dry periods of the year. However, ultrasonic measurements were for most of the time higher than the pressure transducer ones, as shown in Figure 4, especially during the dry period of the year characterized by stable, low flow hydrologic conditions in the stream, with the opposite occurring during periods of the wet season when low flow conditions alternated with higher flow events. As a result, deviations, which were calculated as the percentage differences of pressure stages from the ultrasonic stages, were positive for the greatest part of the time period of the study, which was characterized by relatively high temperatures and low flow conditions, and negative for the shorter cold period of the winter with flow highs. In practice, this can be much related to the operating principle of the ultrasonic sensors, which are sensitive to air temperature. Their sensitivity was however smaller in winter than in summer, in absolute terms, with the higher sensitivity under warm conditions being responsible for the larger deviations of the ultrasonic measurements from the pressure transducer ones. Ultrasonic stages superiority in the warm period was obviously caused by a speed of sound increase under warm atmospheric conditions, which resulted in a small underestimation of the distance from the sensor to the water surface, which was equivalent to a small water stage overestimation (distance from the river bed to the water surface). In this way, and despite any compensation for speed of sound changes, ultrasonic water stages appeared consistently higher than the pressure transducer ones during the warm period of the year, while under the colder conditions of the winter period they appeared to be mostly, but not consistently, lower than the respective measured stages from the pressure transducer.

From the analysis of water stages in parallel with water and ambient temperatures it was shown that the latter was connected with an ultrasonic instability under no sudden hydrologic cause. Diurnal air temperature variations caused the ultrasonic sensor to fluctuate periodically, with stage highs occurring within the day simultaneously with temperature lows, causing the greatest stage deviation from the stage measured by the quite stable pressure transducer. However, even this instability can still be acceptable, since absolute deviations of the ultrasonic sensor from the pressure sensor never exceeded 7%. Especially for the crucial, water stage peaks recorded, deviations were the lowest, with both sensors performing similarly.

A relevant study to the testing of ultrasonic sensors has reported that more than three years of ultrasonic water stage data in streams with normal water stages less than 50 cm in Iowa US, revealed an ultrasonic instability at the level of ±7cm (±15%) on average, with sensors having a strong diurnal cycle behavior [16]. To reduce the error, two methods of compensation were developed. The first substituted the sensor’s internal air temperature compensation using air temperature from a nearby weather station and only reduced the fluctuations by 2 cm on average. The second method however, predicted local air temperature of the stream channel based on the energy balance of the channel and corrected the fluctuations to the order of 1 cm. From these results, it is believed that the 3–4 cm maximum fluctuation range of the ultrasonic sensor at Pikrodafni stream, along with the <7% deviations of the ultrasonic instant measurements from the respective pressure transducer ones did not necessarily require further air temperature compensation. However, a perfect temperature compensation can still be possible for the elimination of the ultrasonic error in the future. Specifically, as a next step to this study, it would be worth investigating the performance of the acoustic compensator in terms of the variability of the speed of sound as a function of the air temperature measured by the sensor and the possible overheating of the sensor and the possible effect on its operation. For the latter, a better temperature measurement would be needed from an external temperature sensor under no direct sun exposure, which would allow for the most accurate temperature compensation, by allowing temperature readings to be taken that better reflect the composite temperature of the acoustic ranging path.

It has to be noted that the automatic monitoring of Greek rivers is a recently started and still ongoing effort, which needs additional time of stations operation to ensure the optimum instruments adjustment, which in turn will ensure the best accuracy of measured data. In particular, for the ultrasonic sensor that is investigated in the present paper, additional time of concurrent measurements with the pressure transducer and the deployment of additional ultrasonic sensors in bigger rivers or even in river sites with higher diurnal temperature fluctuations are needed to support our preliminary conclusions. For example, the use of a certain ultrasonic sensor in this work [30] may have caused inaccuracies in water stage measurements when its internal calibration algorithm works for temperature compensation. Even the sensitivity of measurements to temperature peaks, as shown in Figure 6 and Figure 7, may be partly attributed to excessive calibration of the sensor. Hence, the use of any other sensor of another type could cause slightly different measurements of the water stage due to its particular temperature compensation procedure. This deserves our attention in the future when we will have a larger number of ultrasonic sensors, established in Greek rivers, possibly from different manufacturers, to further judge their accuracy through comparisons.

A recent work has also shown that water level measurements from an ultrasonic sensor can be improved if an algorithm scans the time-series data for removing outliers and random errors such as the ones caused by small waves [36]. From the experience with the Pikrodafni urban stream in the case study of our paper and the relatively short time-series that is already available though, no significant outliers are observed, while errors due to random factors are not expected to be high. However, such an automatic algorithmic process for smoothing the data [36] may exclude the majority of abnormal values in the future when longer ultrasonic time-series data are expected.

From the analysis of this paper, it is concluded that the extensive use of submersible pressure transducers for river stage monitoring, as is the case in the Greek network so far, should be maintained due to the stability and reliability of those sensors. However, even the sensitivity of an ultrasonic sensor to meteorological conditions observed in this study was not significant to indicate a clear inferiority against pressure instruments. Thus, due to the flexibility in their place of installation, non-contact systems such as ultrasonic sensors, present a significant alternative solution in a number of cases, where submersible instruments are prone to gradual wear (e.g., from corrosion), direct damage from debris collision or acts of vandalism. Using a non-contact system not exposed to the constant degradation caused by water and transported materials is always convenient at inaccessible sites and certainly reliable, if accompanied by an accurate air temperature compensation.

## Figures and Tables

**Table 1:** Main statistics calculated for the ultrasonic and pressure water stage data values (*N* = 23,358).

Variable	Mean	StDev	Min	Q1	Median	Q3	Max
Ultrasonic stages (u)	85.220	4.293	78.801	82.801	84.601	86.989	212.976
Pressure stages (p)	83.477	4.163	79.248	81.991	83.210	84.430	221.590
% Deviation (100 × (u − p)/p)	2.0989	1.8741	−5.2639	0.4843	2.6066	3.5947	6.1349

**Table 2:** Pearson correlation coefficients (*r*) between water stages measured by the ultrasonic and the pressure transducer sensors, air temperature and water temperature based on the entire data set available (10-min values within 17 June 2020–27 February 2021).

	Ultrasonic Stage	Pressure Stage	Deviation (%)	Air Temp
Pressure stage	0.935			
Deviation (%)	0.129	−0.229		
Air Temp	−0.096	−0.330	0.670	
Water Temp	−0.062	−0.321	0.737	0.958

## Data Availability

The water stage data from the In-situ AT500 (pressure transducer) instrument and the ultrasonic water stage data are available online at: https://www.openeliot.com/en/ (accessed date: 5 April 2021). The pressure transducer data are also available through the HCMR central online platform at: https://hydro-stations.hcmr.gr/pikrodafni-station/ (accessed date: 5 April 2021) which disseminates the real-time water quantity and quality data recorded from all stations of the Greek monitoring network.
